# Controlling the orientation of nucleobases by dipole moment interaction with graphene/h-BN interfaces

**DOI:** 10.1039/c7ra11664k

**Published:** 2018-02-08

**Authors:** Hakkim Vovusha, Rodrigo G. Amorim, Ralph H. Scheicher, Biplab Sanyal

**Affiliations:** Division of Materials Theory, Department of Physics and Astronomy, Uppsala University Box 516 SE-751 20 Uppsala Sweden ralph.scheicher@physics.uu.se biplab.sanyal@physics.uu.se

## Abstract

The interfaces in 2D hybrids of graphene and h-BN provide interesting possibilities of adsorbing and manipulating atomic and molecular entities. In this paper, with the aid of density functional theory, we demonstrate the adsorption characteristics of DNA nucleobases at different interfaces of 2D hybrid nanoflakes of graphene and h-BN. The interfaces provide stronger binding to the nucleobases in comparison to pure graphene and h-BN nanoflakes. It is also revealed that the individual dipole moments of the nucleobases and nanoflakes dictate the orientation of the nucleobases at the interfaces of the hybrid structures. The results of our study point towards a possible route to selectively control the orientation of individual molecules in biosensors.

## Abstract 2

The interfaces in 2D hybrids of graphene and h-BN provide interesting possibilities of adsorbing and manipulating atomic and molecular entities.

## Graphical abstract

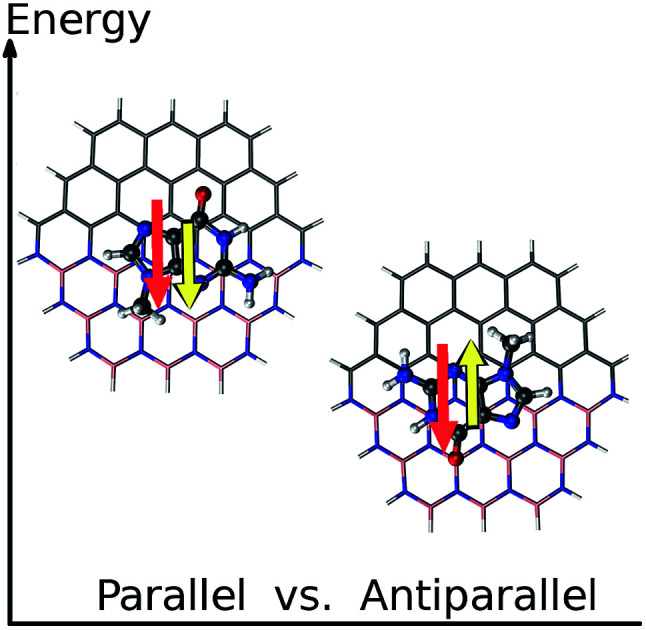

## Introduction

Graphene,^1^ the 2D sp^2^-bonded single layer of graphite, is a hot topic of research today due to its unique electrical,^2–4^ optical,^5,6^ thermal^7,8^ and mechanical properties.^9,10^ An isoelectronic 2D material, namely hexagonal boron nitride (h-BN), has also gained prominence in this field, as its electronic structure contains a large band gap, unlike graphene which exhibits a zero band gap.^11,12^ Both graphene and h-BN have been studied quite extensively in connection to the adsorption of molecules^13^ and nanoclusters^14^ by the π electron cloud.^15^ To understand the self-assembly process of biological molecules like deoxyribonucleic acid (DNA) and ribonucleic acid (RNA),^16–18^ interaction of nucleobases and amino acids with graphene and h-BN have been investigated using experiment and theory. These studies indicate that the noncovalent interactions such as π–π stacking and X–π (X = CH, OH, NH *etc.*) interactions stabilize the nucleobases adsorbed on graphene and h-BN.^19–25^ This shows the potential of using graphene and h-BN in biosensing applications. Moreover, graphene nanoribbons have been proposed to rapidly sequence DNA by measuring the time-dependent conductance as the nucleotides of single-stranded DNA are brought sequentially in contact with the nanoribbon.^26^ Recently Lee *et al.* have studied the interaction of nucleobases with h-BN and graphene using an approach that incorporates van der Waals interactions into the density functional scheme. They found that the binding energies of the different nucleobases with two sheets (h-BN and graphene) are similar and an interfacial dipole is induced between the sheet and base molecule during adsorption.^27^

Very recently, 2D hybrids (CBN) of graphene and h-BN have been prepared through chemical vapor deposition method. These hybrids are found to have unusual electronic, magnetic and transport properties including half-metallicity.^28^ Gao *et al.* have explained the formation of CBN with different edges using Rh (111) substrate and they have shown that formation of zigzag edges is more favorable than that of armchair edges.^29^ The CBN monolayer can be used as an ultrathin solar cell with PCBM fullerene as an acceptor.^30^ Although, the physical properties of these CBN materials have been studied, the interaction of nucleobases with CBN surfaces has not yet been reported. It should be noted that the interaction between the dipoles in the nucleobases and hybrid nanoflakes can give rise to non-trivial properties based on the mutual orientations at the interface of dissimilar flakes. In the present study, we have carried out density functional calculations to investigate different orientations of nucleobases, namely adenine (A), guanine (G), thymine (T) and cytosine (C) adsorbed on CBN nanoflakes with different edges, namely armchair, as well as zigzag with either N- or B-termination. Specifically, we have studied the equilibrium geometries, binding characteristics, and role of dipoles in dictating the mutual orientations.

## Methodology

Structural models of CBN flakes with armchair (Arm) and zigzag (Zig) interfaces were considered from previous studies.^31^ Throughout this article, we refer to interfaces with armchair edges as Arm, to those with zigzag edges where N bonds with C as Zig-N, and to those with zigzag edges where B bonds with C as Zig-B. We have employed *ab initio* calculations based on Density Functional Theory^32,33^ (DFT) and the Linear Combination of Atomic Orbitals (LCAO) as implemented in Gaussian09 ^34^ and SIESTA^35^ codes. We used two levels of calculations to validate our results and give greater confidence in the accuracy of the study: one of them was hybrid meta exchange–correlation functional M06-2X^36^ with 6-31+G(d,p) basis set, while for the second one, we used Generalized Gradient Approximation (GGA-PBE)^37^ with van der Waals corrections^38,39^ to take into account dispersive interactions. For the SIESTA calculations, we considered a box of 30 Å^3^ to avoid the interactions between periodic images. Double-*ζ* polarized basis sets (DZP) and norm-conserving pseudopotentials^40^ were used. To obtain equilibrium structures, forces in three directions on each atom were minimized below 0.01 eV Å^−1^. From here on, meta-GGA is meant to refer to Gaussian09 calculations while GGA + vdW refers to SIESTA calculations.

The stability of the flakes was evaluated using the formation energy as per the following equation:
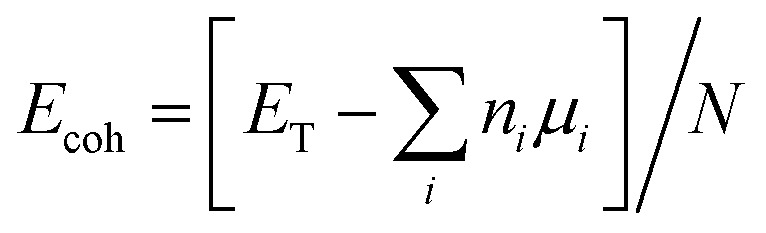
*E*_for_ = [*E*_flake_ − (*E*_GR flake_ − Σ*μ*_C_*N*_C_ + Σ*μ*_B_*N*_B_ + Σ*μ*_N_*N*_N_)]/*N*_tot_

In the first equation, *E*_T_, *n*_*i*_, *μ*_*i*_, *N* refer to: total energy of the flake, number of each species in the flake, corresponding chemical potential, and total number of atoms in the flake, respectively. In the second equation, *E*_flake_ is the total energy of the hybrid flake (Arm, Zig-N, Zig-B). *E*_GR flake_ is the total energy for the graphene reference system, *μ*_*i*_ (*i* = C, N and B) is the chemical potential of each species and *N*_*i*_ (*i* = C, N and B) is the number of extra or missing atoms for a particular species in comparison to the reference graphene flake. *N*_tot_ is the total number of atoms comprising of C, N and B involved in the equation.

## Results and discussion

**Fig. 1:**
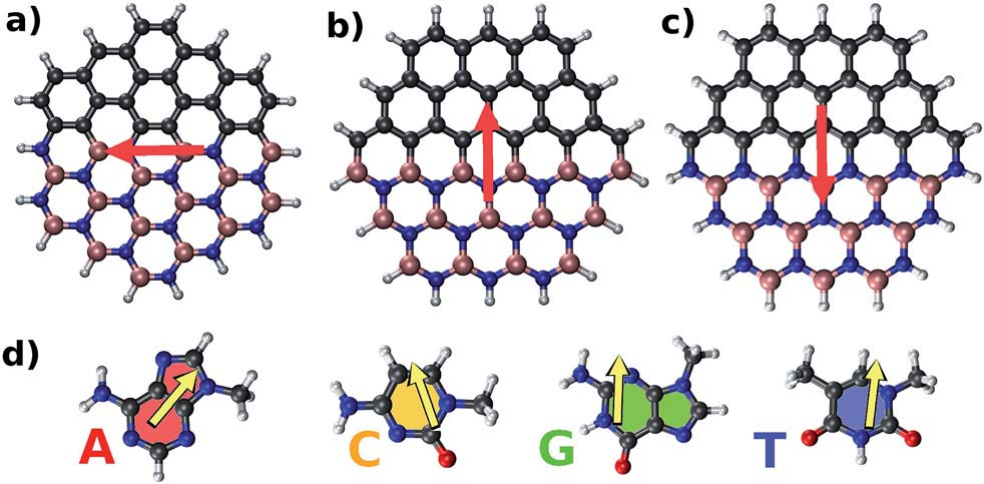
The three different possible interfaces in graphene/h-BN hybrid nanoflakes considered in this study: (a) armchair; (b) zigzag with B–C termination; (c) zigzag with N–C termination. The four nucleobases along with their respective dipole moments are shown in (d): adenine (A), cytosine (C), guanine (G), and thymine (T). Spheres in gray, pink, blue, white and red represent carbon, boron, nitrogen, hydrogen, and oxygen atoms respectively. The red and yellow arrows represent the dipole moment orientations for each flake and nucleobase, respectively, as obtained from our calculations.

Fig. 1 shows structures and dipole moment orientations for the geometry optimized flakes (Arm, Zig-B and Zig-N) and four nucleobases adenine (A), cytosine (C), guanine (G), and thymine (T). We note that due to three possible flake interfaces, the dipole moments have distinct orientations. In Arm, the direction of the dipole moment is along the interface. We note that the total dipole moment (shown in Fig. 1) is formed by the sum of all local dipole moments lying along each BN bond. For the case of Zig-B, the dipole moment is rotated clockwise by 90 degrees compared to Arm flake whereas for Zig-N flake, the direction of the dipole moment is exactly opposite to Zig-B (see Fig. 1(a)–(c)). The dipole moment directions for the nucleobases are shown as yellow arrows in Fig. 1(d). For C, G and T, the arrows point from the oxygen atom (or the center of a pair of oxygen atoms in the case of T) to the opposite side of the nucleobase because the oxygen atom carries excess negative charge. For the nucleobase A, there are three N atoms on one side and two on the other side. The former side has more negative charge than the latter one, which dictates the resulting direction of the dipole moment.

The cohesive energies for the three flakes are given in Table 1, calculated using two different functionals. The results indicate the hierarchy in stability as Zig-N > Arm > Zig-B. The energy differences between Zig-N and Arm (Zig-B) flakes are 6.89 (10.69) kJ mol^−1^ considering GGA + vdW and 3.38 (7.34) kJ mol^−1^ for meta-GGA respectively. The results follow the same trend using both functionals; the cohesive energy difference between the most stable flake (Zig-N) with the least stable (Zig-B) is about two times bigger than that for the second-most stable (Arm) flake. Next, we have calculated the formation energies for the hybrid flakes with GR flake as a reference using the equation given above and the values are 384.32, 473.24, and 479.37 kJ mol^−1^ for the Zig-N, Arm, and Zig-B flake respectively. This result follows the same trend as that of cohesive energies. It should be noted that the positive formation energies indicate that the flakes can be metastable but still possible to form as seen in experiments. The corresponding values are calculated as −457.68, −368.77, −362.63 kJ mol^−1^ when a BN flake is considered as the reference. These values indicate that the hybrid flakes can be spontaneously formed. The difference in these two cases is related to the growth conditions with different reservoirs. As the flake with the B-terminated zigzag edge (Zig-B) is found to be the least stable in all the cases, we will consider only adsorption complexes involving Zig-N and Arm from now on.

**Table 1:** Calculated stability of the three nanoflakes (Arm, Zig-B and Zig-N) using two functionals (GGA + vdW and meta-GGA)

Flake	Cohesive energy (kJ mol^−1^)	
	GGA + vdW	Meta-GGA
Zig-N	−640.07	−689.20
Arm	−633.18	−685.82
Zig-B	−629.38	−681.86

Table 2 lists the total dipole moment values for the two most stable flakes (Zig-N and Arm) and for the four nucleobases, calculated using two different methods. For the nucleobases, we noted the same hierarchy (G > C > T > A) for both functionals and the results are consistent with the previous studies.^41^ For the flakes, we note that Zig-N possesses a total dipole moment about 1.5 to 1.7 times bigger than the Arm flake.

**Table 2:** Calculated dipole moments of the two most stable nanoflakes (Zig-N and Arm) and of the four nucleobases (A, C, G and T) using two functionals (GGA + vdW and meta-GGA)

System	Dipole moment [Debye]	
	GGA + vdW	Meta-GGA
Zig-N	6.12	5.80
Arm	3.61	3.86
G	6.65	6.80
C	5.58	6.01
T	4.37	4.36
A	2.51	2.54

As nucleobases and flakes possess distinct dipole moment values and orientations, an interesting question can be addressed regarding the mutual interaction and the resulting stability of the complexes formed between the nucleobases and the nanoflakes. Specifically the relative orientation between a nucleobase and a nanoflake is of primary interest to us in the present study. To explore this question, we have investigated two fundamentally different geometries for each complex, in which the dipole moments of the constituting subsystems are either parallel or antiparallel to each other.

**Fig. 2:**
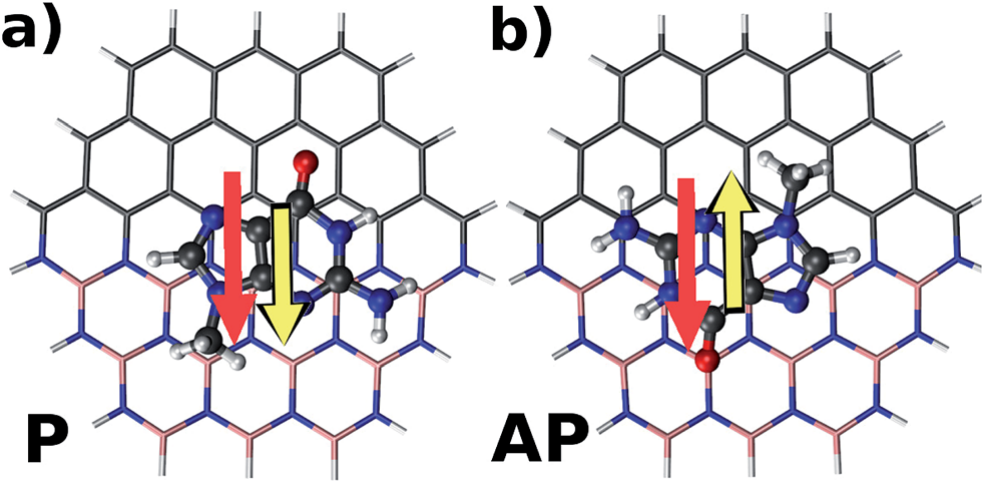
Schematic illustration of a hybrid Zig-N type nanoflake with the nucleobase guanine adsorbed on top of it. The dipole moment of the nanoflake is represented by the red arrow, while the dipole moment of the nucleobase is shown as a yellow arrow. The two distinct relative orientations of the individual dipole moments explored in the present study are: (a) parallel (P) and (b) antiparallel (AP).

Fig. 2 illustrates parallel (P) and antiparallel (AP) dipole moment orientations of the hybrid flakes and a nucleobase, here shown for the example of Zig-N and guanine. The red and yellow arrows indicate the dipole moments of the isolated flake and the nucleobase, respectively. We have performed geometry optimizations of the complexes to find the ground state configurations from which the resulting dipole moments can be extracted. The energy differences between the two orientations (Δ*E* = *E*^P^ − *E*^AP^) can provide insights into the relative stabilities and are listed for all explored combinations of Zig-N and Arm flakes with the four nucleobases in Table 3. As per the above definition, a positive relative energy would indicate that the antiparallel orientation is more stable than the parallel one.

**Table 3:** Calculated relative stabilities of nucleobase–nanoflake complexes with GGA + vdW and meta-GGA

Nucleobase	Stability, Δ*E* = *E*^P^ − *E*^AP^ (kJ mol^−1^)			
	Arm		Zig-N	
	GGA + vdW	Meta-GGA	GGA + vdW	Meta-GGA
G	29.29	12.64	47.89	30.64
C	24.21	19.84	37.26	34.75
T	1.33	2.50	43.16	32.32
A	5.12	0.54	12.67	12.34

Indeed it is found that for all nucleobases on either of the two studied nanoflakes, the antiparallel configuration is always more stable than the parallel one in the Arm and Zig-N flakes, as confirmed by both functionals. However, the nucleobases A and T on Arm are almost degenerate in energy for parallel and antiparallel configurations, indicating no strong preference for either orientation. However, a closer inspection reveals that for GGA + vdW, the antiparallel configuration for A is more stabilized than that for T (5.12 *vs.* 1.33 kJ mol^−1^), whereas in meta-GGA, the reverse is true (0.54 *vs.* 2.50 kJ mol^−1^). This difference may occur due to distinct considerations of dispersion corrections in GGA + vdW and meta-GGA.

As we mentioned above, the nucleobase G possesses the largest dipole moment among the four nucleobases while A carries the smallest. Furthermore, it was also found that the Zig-N flake possesses a larger dipole moment than the Arm one. Now we will analyze the total dipole moments of the complexes with the goal to identify a trend. For complexes with Zig-N, we observe the following order for both methods in the total dipole moment for the parallel (P) arrangement: G > C > T > A. It is interesting to note that these results follow the same hierarchy as that of the isolated nucleobases. For the anti-parallel (AP) orientation, the trend is reversed, *i.e.*, we obtain A > T > C > G. This is due to the fact that in this case the dipole moment gets minimized by partial cancellation of the individual dipole moments (Table 2). The situation is not as straightforward for the Arm flake due to a complex interplay between geometry and charge distribution of the constituents. Hence the resulting total dipole moment cannot be predicted by simple addition or subtraction of the individual dipole moments from Table 2. For the parallel configuration, the hierarchy is G > C > T > A for meta-GGA but G ≈ C > T > A for GGA + vdW. In the energetically more favorable anti-parallel orientation, the sequence order is G > A > T > C for meta-GGA and A > G > T > C for GGA + vdW. These results indicate that full-fledged *ab initio* electronic structure calculations are necessary to provide an accurate picture of the dipole moments of these nucleobase–nanoflake complexes.

**Fig. 3:**
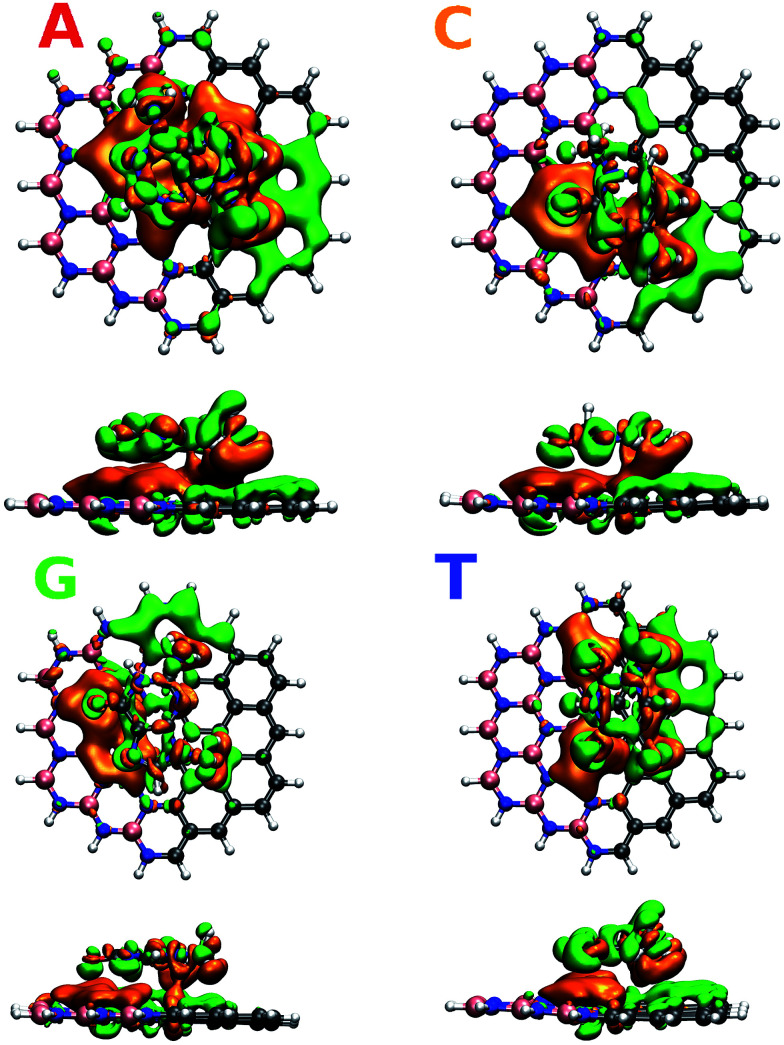
Charge density difference for zigzag N (CBN–ZN) with nucleobases according to the expression given in the text.

To understand in detail the interaction due to adsorption, we calculated charge density differences between the flake-DNA nucleobase complexes and the sum of the charge densities of flakes and DNA nucleobases as described in the following expression:*I* = *I*_flake+DNA_ − (*I*_flake_ + *I*_DNA_),where *I*_flake+DNA_ is the total charge density for the full system and *I*_flake_(*I*_DNA_) is for the flake (DNA), respectively. The charge density difference plots shown in Fig. 3 indicate how the charge is redistributed in the system due to adsorption. The positive charge difference is represented by green and the negative one by orange color. We note that the overall behavior is similar, *i.e.*, the accumulation of positive charge in the graphene side and a negative one in the h-BN side.

**Fig. 4:**
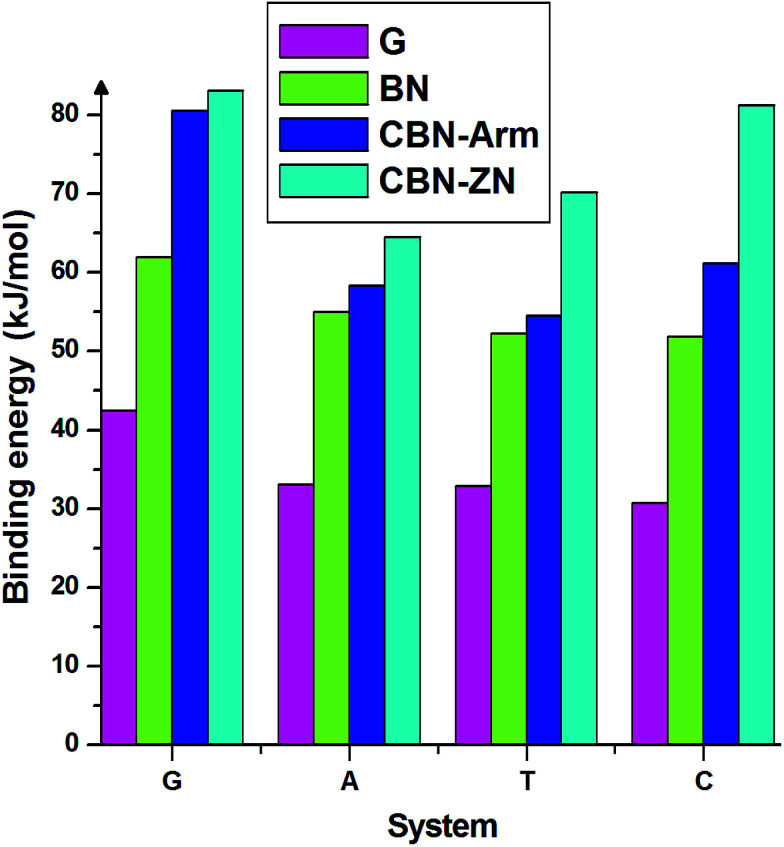
Binding energies of the four nucleobases adsorbed on the nanoflakes made of pure graphene (G), pure boron nitride (BN), armchair (CBN–Arm), and zigzag N (CBN–ZN).

Finally, we discuss the binding energies of the nucleobases on different interfaces of CBN flakes. For comparison, we have also calculated the binding energies on pure graphene and h-BN flakes of the same size as the hybrid systems. These calculations have been carried out using the meta-GGA approach. From the binding energies presented in Fig. 4, it is clear that the hybrid interfaces, especially Zig-N, are better binding agents than the pure flakes for all the nucleobases considered here. It is worth mentioning that the nucleobases have a parallel orientation on the graphene flake whereas a tilted orientation is observed on the BN flake. The calculated dipole moments for pure graphene and BN flakes were 0 and 0.04 Debye and for graphene/h-BN flake, it was 3.85 Debye. Due to the presence of higher dipole moments, nucleobases strongly interact with the graphene/hexagonal BN flake than pure G and BN flakes. This proves the utility of mixed interfaces for stronger adsorption of nucleobases. Moreover, the presence of dipole moments at the interfaces plays an important role in stabilizing certain orientations of the nucleobases.

## Conclusions

In the present work, we have performed first-principles calculations to study the interaction of nucleobases with 2D hybrid nanoflakes formed of graphene and h-BN, considering three different types of interfaces. Our results reveal that the dipole moment orientation of the nucleobases relative to the dipole moment of the hybrid nanoflake interface plays the decisive role in stabilizing these complexes. The antiparallel orientation of dipole moments of the nucleobases with respect to those of the nanoflake interfaces is found to be more stable than the parallel orientation in the armchair and N-terminated zigzag interfaces. Furthermore, the binding energies of the nucleobases on the hybrid flakes are found to be higher than those on both pure graphene and pure boron nitride flakes of the same size. Our study hints at the possibilities of using hybrid flakes to achieve better sensitivity in detecting and identifying specific nucleobases, *e.g.*, for the purpose of DNA sequencing, by improving the signal-to-noise ratio due to the reduction of spatial fluctuations of the nucleobases.

## Conflicts of interest

There are no conflicts to declare.

## Supplementary Material
